# A web‐based multidomain intervention improves modifiable risk factors for Alzheimer's disease

**DOI:** 10.1002/alz70860_103198

**Published:** 2025-12-23

**Authors:** Haakon B. Nygaard, Sylvie Belleville, Nicole D. Anderson, Paul W.H. Brewster, Mohamed Abdelhafid Kadri, Andrew Lim, Manuel Montero‐Odasso, January Durant, Jody‐Lynn Lupo, Penelope J. Slack, John R. Best, Howard Chertkow, Howard H. Feldman, CCNA‐CAN‐THUMBS UP Study Group

**Affiliations:** ^1^ UBC Hospital Clinic for Alzheimer Disease and Related Disorders, Vancouver, BC, Canada; ^2^ Institut Universitaire de Gériatrie de Montréal, Montréal, QC, Canada; ^3^ Ben & Hilda Katz Interprofessional Research Centre in Geriatric and Dementia Care, Toronto, ON, Canada; ^4^ Cognition & Technology Research Group, Institute on Aging and Lifelong Health, University of Victoria, Victoria, BC, Canada; ^5^ Centre de recherche de l’Institut Universitaire de gériatrie du CIUSSS du Centre‐Sud‐de‐l’Île‐de‐Montréal, Montréal, QC, Canada; ^6^ University of Toronto, Toronto, ON, Canada; ^7^ Gait and Brain Laboratory, Canada, ON, Canada; ^8^ University of California San Diego, La Jolla, CA, USA; ^9^ University of British Columbia, Vancouver, BC, Canada; ^10^ Djavad Mowafaghian Centre for Brain Health, Vancouver, BC, Canada; ^11^ Rotman Research Institute, Baycrest Health Sciences, Toronto, ON, Canada; ^12^ Alzheimer's Disease Cooperative Study, Department of Neurosciences, University of California San Diego, La Jolla, CA, USA

## Abstract

**Background:**

The Canadian Therapeutics Platform for Multidomain Interventions to Prevent Dementia (CAN‐THUMBS UP (CTU)) was developed to address modifiable lifestyle risk factors through its online program Brain Health PRO (BHPro), to enable Canadians to participate remotely across a broad geography, and at a significantly lower operational cost compared to in‐person initiatives. This program self surveys multiple risk factors and provides personalized profiles to its participants. We report here on the results of the inception cohort's risk factor profiles and longitudinal outcomes following the BHPro intervention.

**Method:**

A twelve‐month, prospective, multi‐center, longitudinal study of BHPro was launched in March 2022. 353 participants throughout Canada were enrolled, including older adults with no cognitive impairment or those with mild cognitive impairment (MCI), and at least one dementia risk factor (first‐degree family history of dementia, hypertension, hypercholesterolemia, body mass index >30 or physical inactivity). The intervention comprised of 181 online, interactive chapters covering dementia risk factors, delivered progressively over 10 months. The primary outcome was dementia literacy, and secondary outcomes included self‐efficacy, both of which are reported separately. The exploratory outcomes included a change in a composite score of 7 self‐reported, modifiable risk factors, including physical activity, nutrition, cognitive engagement, social/psychological function, vascular health, sleep, and vision/hearing, as well as change in individual risk factors. Lifestyle questionnaires were adapted from published literature.

**Result:**

A covariate‐adjusted mixed linear model for the intention‐to‐treat sample (*N* = 353; mean age = 69.7) revealed a significant positive change in a composite lifestyle risk score at 6 months (standardized change = 0.162; *p* < 0.001), and 12 months (standardized change = 0.167; *p* < 0.001). Individually, physical activity, nutrition, cognitive engagement, and sleep showed statistically significant improvements at both 6 and 12 months.

**Conclusion:**

BHPro, a relatively inexpensive web‐based multidomain intervention, improves key modifiable risk factors for Alzheimer's disease following a program duration of either 6 or 12 months. Future studies will assess efficacy in a larger and more diverse population at risk for Alzheimers disease, and prospectively determine how improving lifestyle may reduce the risk of developing dementia.

